# Bowel Preparation for Pediatric Colonoscopy

**DOI:** 10.3389/fped.2021.705624

**Published:** 2021-09-01

**Authors:** Petar Mamula, Noor Nema

**Affiliations:** Children's Hospital of Philadelphia, Philadelphia, PA, United States

**Keywords:** preparation, pediatric, laxative, colonoscopy, safety

## Abstract

Colonoscopy is an important diagnostic and therapeutic tool in evaluating and treating gastrointestinal tract pathologies. Adequate visualization of the intestinal lumen is necessary for detection of lesions, and thus bowel preparation is a key component of the process. It is estimated that over 25% percent of pediatric patients have sub-optimal bowel preparations, which can lead to longer procedure times, missed pathology, unsuccessful ileal intubation, and possibly repeat procedure/anesthesia. There is no universal protocol for bowel preparation in pediatrics and there is a wide variability of practices around the world. The purpose of this paper is to review the recent published literature regarding bowel preparations for pediatric colonoscopy with focus on published work in the last decade exploring a number of factors involved in bowel preparation including the role of patient education, types of bowel preparation, and their efficacy and safety.

## Introduction

Colonoscopy is an important diagnostic and therapeutic tool in evaluating and treating gastrointestinal (GI) tract pathologies. In pediatrics, common indications include abdominal pain, chronic diarrhea, and hematochezia, and a less common indication includes surveillance for polyposis syndromes. Adequate visualization of the intestinal lumen is necessary for detection of lesions, and thus bowel preparation is a key component of the process. It is estimated that over 25 percent of pediatric patients have sub-optimal bowel preparations (1). This can lead to longer procedure times, missed pathology, unsuccessful ileal intubation (2), and possibly repeat procedure/anesthesia.

There is no universal protocol for bowel preparation in pediatrics, and there is wide variability of practices around the world. These variations include differing laxative agents, duration of preparation, timing of administration, and dietary changes. The purpose of this paper is to review the recent published literature regarding bowel preparations for pediatric colonoscopy, with focus on published work in the last decade. Our group previously reviewed the literature leading up to 2010 and highlighted the vast differences in practices up until that point, and emphasized the need for larger, randomized controlled trials to elucidate a preferred protocol (3). For this current paper, we performed a PUBMED search of all English-language articles relating to pediatric colonoscopy preparation from 2010–2020. This search yielded 13 randomized controlled trials, 9 prospective studies, and 6 retrospective studies (Supplementary Material). These articles explore a number of factors involved in bowel preparation including the role of patient education, types of bowel preparation, and their efficacy and safety. In addition to this search, we reviewed publications relating to technological advances in colonoscopy preparation in adult patients.

## Patient Education

Patient education is an integral part of the bowel preparatory process and lapses of which can impact the quality of the clean out. Identifying institutional risk factors that may lead to poor preparation and gaps in family and patient education should be an area of focus for all hospitals performing pediatric colonoscopy. These risk factors may differ from a center to center. Such risk factors can include poor communication, language barriers, low socio-economic status, and low health literacy. In a retrospective study exploring risk factors for suboptimal bowel preparations, identified risk factors in one center included Spanish-speaking patients and patients with Medicaid insurance coverage (1). In the Spanish-speaking group, one can deduce that the language barrier led to a lapse in patient communication and subsequent understanding of the preparation instructions.

Few pediatric studies have explored improving patient education as means to improving bowel preparation. It is important that patients and their families understand the importance of an adequate clean out and understand the goals of the bowel preparatory process (e.g., to achieve clear stools). A RCT evaluating the impact of an educational cartoon did not show improved Ottawa Bowel Preparation Scale (OBPS) scores in the 20 patients who received the cartoon, but the study did report a positive correlation with education level and quality of bowel preparation (4). A recent study of 42 patients applied the use a Smartphone App to deliver colonoscopy tutorial, instructions, and medication reminders (5). This study showed improved bowel clean out scores in the 20 patients who used the App. Many institutions, including ours, use a multi-pronged approach that employs phone call reminders, emailed instructions, and an animated video to relay instructions to patients and their families. In the current age of technological advances, it will be exciting to see how continued use of such technology can help improve patient education.

## Bowel Preparation Assessment

Reporting adequacy of bowel preparation is an important part of a colonoscopy documentation and allows endoscopists to communicate how well they visualized the bowel. Adequacy of bowel preparation can be assessed by indirect measures such as cecal/terminal intubation rates and procedure duration, but are more accurately assessed by formal scoring systems (6). A number of scales have been developed, with the three most commonly used scales being the Aronchik scale, Ottawa Bowel Preparation Scale, and the Boston Bowel Preparation Scale (BBPS) described in Table 1. The Aronchik scale provides a global assessment of bowel preparation and provides a rating of 1–5, with one indicating an excellent prep characterized by small volume liquid stool and 95% visible mucosa. The Aronchik score is assigned prior to any suctioning/cleaning during procedure. Unlike the Aronchik scale, the OBPS and BBPS rate the bowel preparation by colon segment. In the OBPS, each segment (right colon, mid-colon, and rectosigmoid colon) is given a score of 0–4 and the total colon is given a score of 0–2, with a summative score of 0–14. A score of 0 indicates an excellent prep. This score is also assigned prior to any suctioning. The Aronchik scale and BBPS do not specify what score equates an “adequate” clean out. Lastly, the BBPS provides an assessment of each colonic segment with a score of 0–3, with a summative score of 9. A score of 9 indicates an excellent prep, but a score of ≥ 6 indicates an adequate prep. Unlike the Aronchik scale and OBPS, the BBPS accounts for suctioning and washing. These scoring systems are not validated in pediatrics, but Tutar et al. showed that there is a close correlation (*r* = −0.954) between the OBPS and BBPS scores in a study of 123 pediatric patients (7).

**Table 1:** Bowel preparation assessment scales.

	**Aronchik**	**Ottawa**	**Boston**
Bowel Prep	Provides global assessment of bowel prep	Provides prep assessment by bowel segment	Provides prep assessment by bowel segment
Scoring	1 to 5 (1 = excellent prep)	0 to 14 (0 = excellent prep), 4 points assigned to each segment and 2 points assigned as a total score	0 to 3 (9 = excellent prep)
“Adequate” Prep Score	N/A	N/A	≥ 6
Accounts for Suctioning and Washing	No	No	Yes
Advantages	Easy to apply because it is a global assessment; global assessment may be more applicable in pediatrics where adenoma detection rate (ADR) is not as crucial	Provides assessment by bowel segment	Only scale to provide a defined score for “adequate” assessment. It is a comprehensive scale which provides assessment by bowel segment and accounts for suctioning and washing
Limitations	Does not account for suctioning and washing, does not define a cutoff score for adequate clean-out	Does not account for suctioning and washing, does not define a cutoff score for adequate clean-out, not as easy to apply as Aronchik	Not as easy to apply as Aronchik

The above scoring systems are prone to interpersonal variability, and thus a study in 2020 introduced the use of artificial intelligence (AI) in assessing bowel preparation with a program called ENDOANGEL (8). This AI software was “trained” by reviewing thousands of pre-scored colonoscopy images, using the BBPS. ENDOANGEL provides an assessment of the BBPS during the colonoscope withdrawal phase at an interval of every 30 s. This software was shown to achieve higher accuracy in assessing BPPS as compared to senior endoscopists (8). While this technology is still novel, it provides a promising new objective tool in the assessment of bowel preparation.

## Types of Bowel Preparation

Historically, bowel preparation consisted of whole gut irrigation and lavage, which often resulted in fluid shifts, electrolyte changes, and overall patient discomfort and dissatisfaction. In recent decades, multiple laxative agents have been adapted for use in colonoscopy bowel preparation. Laxatives are categorized by their mechanism of action—osmotic laxatives or stimulant laxatives, but some can have combined effects. Osmotic laxatives are hyperosmolar solutions that typically require large volumes of fluid intake to be effective. Examples include polyethylene glycol (PEG), magnesium-based solutions, and sodium-based preparations. Stimulant laxatives such as sennasoids and bisacodyl are generally more palatable but may cause increased cramping and gastrointestinal discomfort. In this review, we will focus on the most commonly used agents—PEG, senna, and sodium picosulfate preparations (Table 2).

**Table 2:** Commonly used bowel preparations.

**Laxative**	**Sample brand names**	**Mechanism**	**Route of administration and dose**	**Advantages**	**Limitations**
Polyethylene glycol (PEG)	Gialax, GaviLAX, GlycoLax, HealthyLax, MiraLax, PEGyLAX	•Osmotic laxative • Synthetic water-soluble polymer which draws water into the gut and softens the stool	•Comes in powder formulation that is mixed with a sports drink or other form of clear liquid • Dose is 4 g/kg with maximum of 238 g	•Palatable and well-accepted in children • Easily be administered at home	•Requires large volume of fluid • Associated with hypoglycemia in children <7 years of age
Polyethylene with electrolytes (PEG-ELS)	Colyte, GaviLyte, GoLYTELY, NuLYTELY, TriLyte	•Osmotic laxative • Synthetic water-soluble polymer which draws water into the gut and softens the stool	•Can be given by mouth but typically administered via nasogastric tube • Dose is 50–60 ml/kg with maximum of 4 L	•Safe and effective	•Requires large volume of fluid • Not palatable and often requires NG for administration
Polyethylene with electrolytes with ascorbic acid	MoviPrep, Plenvu	•Osmotic laxative • The added ascorbic acid increases the osmolarity of the formulation	•Available in powder and mixed with 50 m/kg of fluid with maximum of 2L • Can be given by mouth or administered via nasogastric tube	•Has higher osmotic effect compared to PEG-ELS, so requires less volume than PEG-ELS	•Limited data in children
Polyethylene with electrolytes with bisacodyl	GaviLyte-H and Bisacodyl	•Osmotic and stimulant properties • Bisacodyl stimulates the parasympathetic nervous system in the colon	•PO • Comes in 2L bottle of Gavilyte with one 5 mg Bisacodyl delayed-release tablet	•Requires less volume than standard PEG-ELS or PEG	•Not palatable
Sodium picosulfate/magnesium oxide/citric acid (SMPC)	Clenpiq, CitraFleet, PicoLax, Picoprep	•Combination medicine with stimulant and laxative properties • Sodium picosulfate is a prodrug that is metabolized into gut bacteria and causes peristalsis • Magnesium citrate and citric acid are osmotic agents	•Comes in 100 g powder sachets and ready-to-drink formulations. The powder is designed to be mixed in 150–250 ml of fluid. Most regimens call for 2 doses.	•Lowest volume preparation available • Palatable	•Not approved for children younger than 9 years old in many countries
Sodium sulfate/potassium sulfate/magnesium sulfate	Suprep	•Osmotic laxative • Sulfate salts are poorly absorbed and draw water into the gut	•Package comes in 2 liquid bottles, each of which is diluted in 360 mL	•Lower volume than PEG preparations	•Not approved in children younger than 12 years old
Senna	Sennakot, ExLax, Lax Pills	•Stimulant laxative • It is an anthraquinone plant derivative which increases colonic transit	•Comes in oral liquid, pills, or chewable tablets • Dose is 3 mg/kg/d in 2 divided doses, with maximum dose of 150 mg/d. • If used in conjunction with PEG, dose is typically 26.4 mg (15 ml) for children ages 6–12 or 52.8 mg (30 ml) for children > 12 years	•Easy to administer	•Not effective in bowel cleansing as monotherapy • Can cause cramping and abdominal pain

### Polyethylene Glycol

Polyethylene glycol (PEG) is one of the most commonly used agents for bowel preparation in both adults and children worldwide. It is a synthetic water-soluble polymer which functions by drawing water into the gut and softening the stool. PEG exists in multiple formulations—with primary distinction being PEG with and without electrolytes. PEG may also have additives such as ascorbic acid and bisacodyl.

PEG with electrolytes (PEG-ELS) is a salty unpalatable solution that often requires administration via nasogastric tube in children (9). PEG-ELS is given in large volumes with doses up to 25 mL/kg/hr, with a maximum volume of 4 liters. PEG-ELS has been shown to be efficacious and safe and is widely used around the world. Studies have shown an adequate bowel cleansing rate of 88.4% (10). Oral intake of PEG-ELS has been falling out of favor in pediatrics due to difficulty of administration and taste. In a small study of 35 patients receiving PEG-ELS, up to 77.1% of patients rated the taste as “very bad” and 57.1% of patients rated the bowel preparation as “very difficult” (11). Similar results were shown in other studies (9, 10, 12). Newer PEG-ELS preparations now contain ascorbic acid; this is more palatable and has a higher osmotic effect, allowing for half the required volume. A pilot retrospective study showed this to be an effective regimen in pediatrics, though these patients also received a dose of sodium picosulfate (13).

PEG 3350 without electrolytes (e.g., Miralax®, Bayer Healthcare, Whippany, NJ), originally used for management of constipation and fecal impaction, is now the most commonly used bowel preparation. It has become increasingly popular as it comes in a tasteless powder form that can be dissolved in clear liquid or sports beverage. Similar to PEG-ELS, PEG without electrolytes also requires large volume of fluid intake. Thus, many protocols call for combination regimens with a stimulant such as senna or bisacodyl with lower volumes of liquid. Earlier regimens of PEG 3,350 without electrolytes called for protocols as long as 4 days, but shorter regimens of 1–2 days have been shown to be effective and tolerable (14–17). Phatak et al. showed that 92–93% of 111 pediatric patients receiving 2 days of PEG with bisacodyl achieved “good” or “excellent” bowel preparation (18). A large two-part retrospective and prospective study of 656 patients on 1 day of oral PEG-3350 monotherapy reported adequate clean out (defined as thin or thick liquids) in 79.5 and 15.8% of cases, respectively (19). While the safety of PEG without electrolytes has been questioned, two studies reviewing electrolytes pre and post PEG-3350 did not show clinically significant changes in potassium or bicarbonate (17, 20). However, Sahn et al. did report a risk of hypoglycemia in patients younger than 7 years old (20). Thus, it is our practice to obtain glucose serum levels for all patients immediately prior to undergoing colonoscopy.

A meta-analysis of randomized controlled trials (RCT) in adult cohorts showed that Miralax® and Gatorade® (PepsiCo, Chicago, IL) is inferior to PEG-ELS (21). While head-to-head data in pediatrics is limited, a study comparing PEG-ELS and PEG without electrolytes + bisacodyl showed similar efficacy in both groups (88.4 vs. 87.8% respectively) and importantly showed increased acceptability and tolerability in the latter group (10, 11). Nausea and vomiting are common adverse effects associated with both PEG-ELS and PEG without electrolytes, but these side effects can be ameliorated with anti-emetics. A RCT of 308 adult patients receiving PEG for bowel preparation found that D2 receptor antagonists (domperidone and sulpiride) were associated with less abdominal discomfort. Similar studies are needed in pediatrics cohorts, especially in the context of increasing use of PEG without electrolytes (22).

### Sodium-Based Preparations

Sodium-based preparations are lower-volume osmotic laxative, introduced as gentler alternatives to PEG preparations. Earlier formulations with sodium phosphate were shown to be associated with hyperphosphatemia and higher risk of nephrotoxicity (acute kidney injury and chronic tubular injury). In fact, the Food and Drug Administration (FDA) has recommended avoidance of oral sodium phosphate in patients younger than 18 years and has issued a black box warning (23). Additionally, sodium phosphate was shown to distort colonic mucosa and cause aphthoid lesions, and is thus contraindicated in patients undergoing colonoscopy for IBD evaluation (24).

Subsequent sodium formulations such as sodium sulfate and sodium picosulfate have shown to be safe alternatives to sodium phosphate, and lower-volume, equally efficacious alternatives to PEG (10–12, 25, 26). Sodium picosulfate can be administered alone but is also given as a combination medication with magnesium oxide and citric acid. A randomized controlled trial (RCT) of 72 pediatric patients comparing PEG-ELS (25 mg/kg/h) with sodium picosulfate (100 g ×2 doses) showed no difference in bowel preparation between the two groups, but did show sodium picosulfate to be more tolerable in terms of taste and ease of administration (11). Eighty percent (28/35) of patients receiving sodium picosulfate regimen rated the taste as “good” or “very good” as opposed to none in the PEG group (11). Differences in tolerability are not as drastic when comparing sodium picosulfate to PEG without electrolytes but were still statistically significant. A trial comparing three regimens: (1) PEG-ELS, (2) PEG without electrolytes, and (3) sodium picosulfate + magnesium oxide + citric acid (SMPC) showed the highest acceptability in the SMPC group, followed by PEG without electrolytes (10). Bowel preparation was equally efficacious in all three groups. A large RCT of 288 patients in Italy compared three different PEG regimens (PEG-ELS, PEG with citrate and bisacodyl, and PEG with ascorbic acid) with SMPC and recapitulated similar findings (26). Successful bowel preparation, defined as BBPS ≥ 6, was similar in all 4 groups (83.3–91.7%,) with no statistical difference. As in prior studies, the rate of children willing to repeat the same preparation was significantly higher in the SMPC group. Side effects including nausea, bloating, and abdominal pain were also significantly lower in the SMPC group. Lastly, it is important to note that this study included safety outcomes and found no significant differences in electrolyte levels (pre and post procedure) between all four groups.

### Senna/Sennosides

Senna is anthraquinone plant derivative which acts as a stimulating laxative when orally ingested. It is not systemically absorbed and it is degraded into its active metabolite in the lower GI tract which subsequently increases colonic transit (27). Like other stimulant laxatives, side effects include abdominal cramping and nausea. Senna is typically used in combination with an osmotic laxative in bowel preparation, as studies have not shown it to be consistently efficacious as monotherapy. Our group conducted a RCT comparing 2 days of senna with oral PEG-3350 and showed that senna was far inferior, with only 29% of patients achieving adequate bowel clean out as opposed to 88% in the PEG group (28). This study was prematurely stopped as the senna regimen was insufficient for bowel preparation. Conversely, a recent RCT showed similar efficacy between pediatric patients receiving senna for 3 days and PEG3350 with bisacodyl (7). However, patients who received the Senna were less satisfied with the process and less willing to repeat the preparation (7). The Senna group was restricted to a full liquid diet for 2 days followed by 1 day of clear liquid diet (CLD), whereas the PEG group was only restricted to 1 day of CLD. Most recently, a study in India evaluated a combination product of senna and probiotic (Bacillus coagulans)(M Sip Lax® straws, Inzpera Healthsciences Ltd, Mumbai, India) with rectal enema and found that 93% (28/30) patients achieved an adequate bowel clean out, defined as BBPS of 3 in each segment (29). This group postulated that the probiotic provided a synergetic effect with the senna by promoting water absorption into the colon.

### Other Stimulants

Other stimulant laxatives such as bisacodyl are often used as adjunctive agents to osmotic laxatives for bowel preparation. Bisacodyl works by stimulating the enteric neurons to generate peristalsis. Similarly, non-pharmacologic approaches such as gum chewing have been postulated to have similar effects on the parasympathetic pathway by stimulating the vagal nerve and subsequently promoting GI tract motility. There is limited data on gum chewing, although a RCT of 300 patients did not show any differences in bowel cleansing between the group who was instructed to chew gum vs. the control group (30). However, gum chewing improved patient satisfaction.

## Timing/Administration of Bowel Preparation

In addition to choosing an appropriate laxative agent, it is important to consider how timing and administration of such medications can impact bowel preparation. With respect to PEG 3350, it has been observed that consumption of bowel preparation over a shorter period of time is associated with a better bowel cleanout (17). In a prospective study of 45 patients receiving PEG3350 with Gatorade, patients who had “excellent” or “good” bowel preparations consumed the prescribed regimen in a shorter period of time, whereas the patient who had a “poor” preparation required 8.5 h to ingest the solution (17). This finding was not statistically significant but raises an interesting question regarding rapid administration of an osmotic laxative.

Split-dose PEG regimens, which have become a standard in many adult institutions, should also be taken into consideration. Under this regimen, half the prescribed volume of bowel preparation is given the evening prior to colonoscopy and the second half is given on the morning of the procedure. Split-dose PEG regimens have been shown to be more effective than single-dose regimens in adults (31, 32). This finding is attributed to the decreased duration between laxative and procedure time, and is attributed to improved compliance (32). Until very recently, split-dose regimens have not been attempted in pediatric patients, as there are limitations with NPO times and implementing split-dose regimens in the early morning. A trial of 179 pediatric patients comparing split-dose PEG with a single-dose PEG showed the split-dose to be more tolerable and more effective (33). The patients in the split-dose group received the first dose between 6:00–8:00 PM in the evening prior to colonoscopy and then at 6:00–8:00 AM in the morning of the procedure. Colonoscopy was scheduled in the afternoon. Surprisingly, patients reported less sleep disturbance with split-dose regimen. A second RCT of 45 pediatric patients also showed superior efficacy, acceptability, and decreased side effects in the split-dose group (34).

## Dietary Changes During Bowel Preparation

Most pediatric bowel preparations recommend a clear liquid diet on the day prior to procedure. However, many groups are questioning the necessity of implementing a clear liquid diet, as opposed to a low residue/fiber diet. A low residue diet is more flexible and allows for consumption of dairy products, meats, pasta, and some breads. Multiple meta-analyses in adult cohorts have shown that the adequacy of bowel preparation is similar between patients on clear liquid diet and those on a low residue diet (35, 36). Based on this evidence, the European Society of Gastrointestinal Endoscopy (ESGE) 2019 guidelines strongly recommended the use of a low residue diet for bowel preparation (37). Though pediatric data on this subject is limited, a recent randomized controlled trial of 184 patients in Poland found no significant difference in BBPS between patients on a clear liquid diet and patients on low residue diet on the day prior to procedure. Both groups of patients received PEG-ELS (38). Further studies are needed to evaluate whether a low residue diet is appropriate with regimens other than PEG-ELS. This is especially important in pediatrics, where dietary restrictions are likely to cause greater disturbance in daily life and may lead to reduced compliance with the overall bowel regimen.

## Bowel Cleansing Devices

In addition to optimizing oral preparations, there have been new efforts to develop bowel cleansing devices that can be used intra-procedurally or prior to procedure. The FDA recently cleared the Pure-Vu® system (Motus GI Holdings, Fort Lauderdale, FL) for use of bowel cleansing in poorly prepped colons. The Pure-Vu® system is a disposable sleeve that is attached to the colonoscope and uses a vortex mixture of water and air to break up fecal matter. Like a standard flush pump, the endoscopist uses a foot pedal to activate the device. Three recent studies have shown that the Pure-Vu system is successful in improving bowel preparation quality (39–41). Patients in these studies only received Bisacodyl prior to the procedure. It is important to note that these pilot studies excluded patients with inflammatory bowel disease, which is one of the more common indications for colonoscopy in children. Further studies are needed to assess safety of this device in patients with active inflammation.

Unlike the intra-procedural Pure-Vu system, the HyGIeaCare® (Lifestream Purification Systems, LLC) is a novel system designed to assist in bowel preparation prior to colonoscopy. Patients who undergo this preparation are seated in private sanitized basin and then have a disposable nozzle introduced in the rectum. This nozzle infuses a steady stream of warm water to help break up the stool. This process is intended to take less than an hour and eliminates the need for multiple bathroom trips. While this is less disruptive than traditional oral cleanout, we question whether children would be able to tolerate the process.

## Summary

An ideal bowel preparation is one that is efficacious, safe, palatable, and minimally disruptive to a patient's daily life. While no current bowel regimen meets all such criteria, there are multiple safe and efficacious one-day regimens in use. In the last decade, PEG without electrolytes has become increasingly popular in the United States; and more recently sodium picosulfate formulations have begun to gain traction around the world. The data suggests that both regimens are equally efficacious, but sodium picosulfate is more accepted and tolerable to patients. Larger randomized controlled trials are needed to compare efficacy and safety of these two preparations.

The wide variation in bowel cleansing regimens serves as an advantage to our patients and allows for an individualized approach based on a child's specific needs and abilities. PEG-ELS is an ideal option for patients with a nasogastric or gastrostomy tube at baseline, as the feeding tube eliminates the discomfort related to the large volume and poor palatability with PEG-ELS. Sodium picosulfate preparations are a good option for children who have trouble tolerating large volumes of fluid. PEG without electrolytes is a good option for children who are willing to drink larger volumes of liquid that is flavored with their beverage of choice. While most institutions implement a standard bowel cleansing protocol, practices may shift to involve a more patient-centered approach.

Other factors of bowel preparation such as split-dosing regimens, dietary restrictions, and use of technology in patient education remain an area that should be further explored in pediatrics. Exploring the role of a low residue diet may be especially impactful in pediatric patients as this will make the bowel cleansing process more tolerable.

## Author Contributions

All authors listed have made a substantial, direct and intellectual contribution to the work, and approved it for publication.

## Conflict of Interest

The authors declare that the research was conducted in the absence of any commercial or financial relationships that could be construed as a potential conflict of interest.

## Publisher's Note

All claims expressed in this article are solely those of the authors and do not necessarily represent those of their affiliated organizations, or those of the publisher, the editors and the reviewers. Any product that may be evaluated in this article, or claim that may be made by its manufacturer, is not guaranteed or endorsed by the publisher.
