# Supplementary material for: Bowel Preparation for Pediatric Colonoscopy
Source: Front Pediatr. 2021 Sep 1;9:705624. doi: 10.3389/fped.2021.705624 (PMC8442953; doi:10.3389/fped.2021.705624)
Supplement: Supplementary file 1 [file Table_1.docx]

**Supplementary Table**

| **Pediatric Colonoscopy Preparation Studies from 2010-2020** | | | | |
| --- | --- | --- | --- | --- |
| **Paper** | **Study Design** | **N** | **Description** | **Study Conclusion** |
| Vejzovic et al. | Randomized controlled trial | 70 | Compared PEG-ELS (25 ml/kg/h) with 2d sodium picosulfate, used OBBS to assess preparation | No difference in bowel preparation between the 2 groups, but sodium picosulfate was found to be more tolerable |
| Szaflarska-Poplawska A et al. | Randomized controlled trial | 123 | Compared 3 regimens – 1) PEG without electrolytes, 2) PEG-ELS, and 3) sodium picosulfate/magnesium oxide, and citric acid, used BBPS to assess preparation | No difference in bowel preparation between the 3 groups; acceptability was highest in sodium picosulfate group followed by PEG without electrolytes |
| Elitsur R et al. | Prospective | 78 | Compared 4d PEG-ELS vs 2d PEG-ELS + bisacodyl regimen | No difference in bowel preparation between the 2 groups, but the 2d regimen considered superior due to shorter length |
| Elitsur R et al. | Retrospective | 89 | Studied efficacy of 2d PEG-ELS + bisacodyl regimen | 2d PEG-ELS + bisacodyl regimen is effective in achieving adequate bowel preparation |
| Tsunoda T et al. | Retrospective | 112 | Studied safety and feasibility of lower-volume PEG-ELS regimen with ascorbic acid + 1 dose sodium picosulfate | Lower-volume PEG-ELS with ascorbic acid is effective in achieving adequate bowel preparation |
| Phatak UP et al. | Prospective | 111 | Studied efficacy of 2d PEG-3350 + bisacodyl regimen | 2d PEG-3350 regimen is safe, effective, and well accepted |
| Abbas et al. | Prospective, open label | 46 | Studied efficacy of 1d PEG-3350 mixed in Gatorade, used BPPS scale | 1d PEG-3350/Gatorade regimen is effective in achieving adequate bowel preparation |
| Walia et al. | Prospective | 45 | Studied efficacy, safety, and tolerability of 1d PEG 3350 mixed in Gatorade, used BPPS scale | 1d PEG-3350/Gatorade regimen is effective, safe, and tolerable. Patients noted to have lower bicarbonate but did not have features of metabolic acidosis |
| Sahn B et al. | Randomized controlled trial | 155 | Tested safety of 1d PEG-3350, compared electrolytes pre and post cleanout | 1d PEG-3350 is safe; children younger than 7 years of age are at risk for hypoglycemia |
| Terry NA et al. | Randomized controlled trial | 30 | Compared 2d Senna vs PEG-3350, used Aron chick scale | Study prematurely stopped because senna group was inferior to the PEG3350 regimen in bowel preparation |
| Tutar E et al. | Randomized controlled trial | 128 | Compared 1d PEG-3350 + bisacodyl vs 3d senna, used OBPS and BBPS to assess preparation | Similar efficacy in both groups, but PEG-3350 was better accepted. Also noted high correlation between OBPS and BBPS |
| Kumar AS et al. | Retrospective and prospective (two-part study) | 656 | Studied efficacy of 1d PEG-3350 monotherapy | 1d PEG-3350 monotherapy is effective in achieving adequate bowel preparation |
| Jibaly R et al. | Prospective | 30 | Studied efficacy of 2d PEG-3350 monotherapy, used Aronchick scale | 2d PEG-3350 monotherapy is effective in achieving adequate bowel preparation |
| Di Nardo G et al. | Randomized controlled trial | 299 | Compared 4 regimens: 1) PEG-ELS, 2) PEG-ELS with citrate + bisacodyl, 3) PEG-ELS with ascorbic acid, 4) 2 doses sodium picosulfate/magnesium oxide/citric acid, used BBPS to assess preparation | Similar efficacy in all 4 groups |
| Cuffari C et al. | Randomized controlled trial | 78 | Compared oral PEG vs sodium picosulfate/magnesium oxide/citric acid (SMPC), used Aronchik scale to assess preparation | Similar efficacy in both groups, but the SMPC found to be more tolerable |
| Cisarò et al. | Prospective | 50 | Studied efficacy of sodium picosulfate-magnesium citrate (SPMC), used BBPS to assess preparation | SMPC is an effective in achieving adequate bowel preparation |
| Sriphongphankul H et al. | Randomized controlled trial | 45 | Compared single-dose PEG-ELS regimen to split-dose regimen | Bowel preparation was better in split-dose group |
| Tripathi et al. | Randomized controlled trial | 179 | Compared single-dose PEG-ELS regimen to split-dose regimen | Bowel preparation was better in split-dose group. Split-dose group also reported less sleep disturbance |
| Kesavelu D Sr et al. | Prospective | 30 | Studied efficacy of senna and probiotic-based preparation, used BBPS to assess preparation | Successful bowel preparation observed in all patients |
| Reddy P et al. | Retrospective | 908 | Evaluated risk factors for sub-optimal bowel preparation, no standard prep used in all patients, used Aronchik scale to assess preparation | Identified risk factors for poor preparation included younger age patients, Spanish-speaking patients, Medicaid patients, and those with failure to thrive |
| Brief J et al. | Randomized controlled trial | 42 | Evaluated impact of a Smartphone App on bowel preparation, used BBPs to assess preparation | Bowel preparation scores were higher in the group who used the smartphone app |
| Mytyk A el al. | Randomized controlled trial | 184 | Compared clear liquid diet to low residue diet in patients receiving PEG-ELS, used BBPS to assess preparation | No significant difference in bowel preparation between the 2 groups, noted correlation between drinking time of PEG-ELS + BBPS score |
| Maxwell E et al. | Randomized controlled trial | 23 | Studied impact of instructional cartoon on bowel preparation in patients receiving PEG-3350 + bisacodyl, used OBPS to assess preparation | No significant difference in bowel preparation between the 2 groups, noted positive correlation with education level and OBPS |
| Berger T et al. | Open observational study | 768 | Reviewed characteristics of 768 colonoscopies | Reported that 2 most commonly used regimens are sodium picosulfate and PEG. Patients receiving PEG more likely to require NG tube. Majority of bowel preparations were 1-day preparations. |
| Pasquarella CS et al. | Retrospective | 391 | Studied the ileal and cecal intubation rate in a single center and evaluated the factors that contributed to successful intubation | Poor bowel preparation accounted for failed cecal intubation in 23% of patients |
| Yoshioka S et al. | Retrospective | 110 | Summarized characteristics and diagnoses of patients undergoing colonoscopy in Japan. Also evaluated types of bowel preparation and types of sedation used. | Study found that most patients <12 years received magnesium citrate and patients >12 years received PEG-ELS |
| Hart et al. | Prospective | 11 | Qualitative study to determine barriers and facilitators to good bowel preparation in patients receiving sodium-picosulfate, magnesium oxide and citric acid | Patients reported confusion regarding mixing of the preparation and as well as lack of clarity on expected goals in terms of rectal effluent |
| Fang et al. | Randomized controlled trial | 150 | Compared 2 split-dose regimens of PEG-ELS (40 ml/kg vs 60 ml/kg) in inpatients | No significant difference in bowel preparation between both groups |
